# Assessment of Lung Function and Its Correlation With Iron Overload in Children With Thalassemia Major

**DOI:** 10.7759/cureus.87278

**Published:** 2025-07-04

**Authors:** Rufeena J, Upendra P Sahu, Shambhavi Datta, Prity K Rajak, Praveen Kumar Singh

**Affiliations:** 1 Paediatrics, Rajendra Institute of Medical Sciences, Ranchi, Ranchi, IND; 2 Paediatric Medicine, Rajendra Institute of Medical Sciences, Ranchi, Ranchi, IND

**Keywords:** iron overload, lung dysfunction, serum ferritin, spirometry, thalassemia major

## Abstract

Introduction: Thalassemia major is a genetic disorder characterised by defective red cell production. Due to the requirement of regular blood transfusions, these patients are at risk of iron overload, leading to iron deposition in all organs, including the lungs, causing hemosiderosis of the lung, thereby causing impairment of lung function.

Objectives: The primary objective was to assess lung dysfunction in children with thalassemia major and to correlate it with serum iron status. The secondary objectives were to find the prevalence, type and severity of lung dysfunction in these children.

Methodology: A cross-sectional study was conducted in the paediatrics department of a tertiary care hospital in Jharkhand, Eastern India, from December 2022 to April 2024. Eighty children with a diagnosis of thalassemia major, aged 6 to 18 years, with a history of more than 10 blood transfusions, were enrolled. Forced expiratory volume in one second (FEV1), forced vital capacity (FVC), and FEV1/FVC ratio estimation by spirometer, serum ferritin and CRP were assessed.

Results: Of the 80 children included in the study, 52 were males (65%) and 28 were females (35%). A total of 87.5% (n = 70) of them showed a restrictive pattern of lung involvement. Around 52.5% (n = 42) had mild, 18.75% (n = 15) had moderate, 10% (n = 8) had moderately severe, and 6.25% (n = 5) had severe restriction of lung function. The mean serum ferritin values were 3344.55 ng/mL, 5657.07 ng/mL, 5165.5 ng/mL and 8024.2 ng/mL in children with mild, moderate, moderately severe and very severe restriction of pulmonary function, respectively. The serum ferritin levels had a significant correlation with lung function (r = -0.78, p = 0.001).

Conclusion: In this study, we observed varying severity of restrictive type of lung dysfunction in children with thalassemia major despite regular iron chelation. There was a statistically significant association between varying severity of lung impairment and body iron status as measured by serum ferritin levels.

## Introduction

Beta thalassemia major is a genetic disorder characterised by defective red cell production in bone marrow, leading to impairment of oxygen supply to the tissues. Thalassemia major is responsible for most of the cases of severe thalassemia worldwide [1]. Due to the requirement of regular blood transfusions, these patients are at risk of iron overload, which, despite adequate chelation therapy, eventually occurs. In thalassemia major, repeated blood transfusions lead to iron deposition in all organs, including the lungs, leading to hemosiderosis of the lung, thereby causing impairment of lung function [2]. Current studies show that a larger population of thalassemia patients are developing lung dysfunction as a major sequela to iron overload [3,4].

The mechanisms of lung injury in thalassemia are hemosiderosis of the lung due to repeated transfusions, pulmonary hypertension and cardiac dysfunction due to myocardial iron deposition. A restrictive type of lung dysfunction has been reported in most of the studies [5,6], while few studies have also reported obstructive [4] and diffusional dysfunction [7,8]. Monitoring of iron overload status in patients with thalassemia is necessary. In these patients who require chronic blood transfusions, serum levels of ferritin play an important role in guiding the levels of body iron [9].

The rationale behind this study is that though a restrictive type of lung dysfunction has been reported in most of the studies, research data on this topic from eastern India, which has a high prevalence of thalassemia, is scarce [10]. Moreover, recent studies suggest that early diffusional impairment in transfusion-dependent thalassemia can be reversed by intensive chelation therapy [11].

Hence, if a correlation of pulmonary function impairment and iron overload status can be established, regular screening of these patients for lung impairment can be done, and if detected earlier, these patients can benefit from intensive chelation therapy.

## Materials and methods

This study was conducted in the paediatrics department of a tertiary care hospital, Rajendra Institute of Medical Sciences, Ranchi, Ranchi, in Jharkhand, a state in Eastern India, as a cross-sectional study. Upholding all the ethical standards, and after receiving Institutional Ethical Committee approval, the study was undertaken from December 2022 to April 2024.

The study participants included children aged 6 to 18 years with β-thalassemia with a history of more than 10 blood transfusions, admitted for blood transfusion after getting informed consent from their parents and assent from the children. The children who were excluded from the study were those who were not willing to participate in the study, children unable to perform spirometry and children with known chronic lung diseases.

After collection of basic demographic data, detailed history including the age at diagnosis, duration of the disease, age at the start of blood transfusion, frequency of transfusion, details regarding intake of iron chelators were noted, followed by general physical and systemic examination of all participants of the study. We performed a pulmonary function test (PFT) using a spirometer and assessment of serum ferritin and serum C-reactive protein. All the data was collected using a case record form. As inflammation causes variation in serum ferritin values independent of iron overload status, C-reactive protein levels were measured in order to rule out false-positive elevation of levels of serum ferritin levels. The participants underwent spirometry to assess pulmonary function and serum ferritin estimation for iron overload.

The outcomes measured were lung function tests (LFTs) using spirometry, including forced vital capacity (FVC), forced expiratory volume in one second (FEV1) and ratio of FEV1/FVC, and body iron status by serum ferritin. The type of lung dysfunction was categorised into restrictive and obstructive lung dysfunction by the FEV1/FVC ratio. A ratio of more than 70% with the total lung capacity (TLC) less than 80% suggests a restrictive type, while a ratio of less than 70% suggests an obstructive type of lung dysfunction [12]. The restrictive type of lung dysfunction can be further graded in severity as mild when the ratio is more than 70%, moderate is 60% to 69%, moderately severe is 50% to 59%, severe is 35% to 49% and very severe when it is less than 35% of the predicted value as per the severity grading of American Thoracic Society [12,13].

The data collected was analyzed to find the incidence of lung dysfunction, the type of lung dysfunction either obstructive or restrictive using the FVC, FEV1 and FEV1/FVC ratio, and the extent or severity of lung involvement as mild, moderate, severe and very severe disease, and its correlation with serum ferritin levels was also analyzed. Statistical analysis was done using IBM SPSS Statistics for Windows, Version 20 (Released 2012; IBM Corp., Armonk, New York, United States) and the values were reported as mean ± standard deviation (SD).

## Results

The study included 100 children, of whom 20 were lost during the study duration. Hence, the total sample taken for the analysis was 80. Of the 80 children included in the study, 28 were females and 52 were males. The mean age of the children included was 10.2 years. The mean age at the start of blood transfusion was 7.3 months, with the mean total blood transfusion of 114.6 transfusions. All the participants of this study were on regular oral iron chelation with deferasirox, with the mean age at the start of chelation being 4.1 years and mean pre-transfusion haemoglobin of 5.48 g/dL as depicted in Table 1.

**Table 1:** Baseline characteristics of children with thalassemia major included in the study

Variable (N = 80)	Mean ± SD
Male (n)	52
Female (n)	28
Age (years)	10.28 ± 2.819
Height (cm)	124.19 ± 12.225
Weight (kg)	24.45 ± 6.531
Age at start of blood transfusion (months)	7.3 ± 2.79
Age at start of chelation (years)	4.13 ± 0.96
Total number of blood transfusions	114.66 ± 47.737
Pre-transfusion haemoglobin (g/dL)	5.48 ± 0.737

The restrictive type of lung involvement was seen amongst all age groups of thalassemia major patients. In this study of 80 participants, 52.5% (n = 42) had mild restriction, 18.75% (n = 15) had moderate restriction, 10% (n = 8) had moderately severe restriction, and 6.25% (n = 5) had severe restriction of lung function (Table 2).

**Table 2:** Comparison of severity of restrictive lung dysfunction and serum ferritin levels in children with thalassemia major (N = 80) FEV1: forced expiratory volume in one second; FVC: forced vital capacity; SD: standard deviation

Severity of restrictive lung disease	Number of participants	FEV1% (mean ± SD)	FVC% (mean ± SD)	FEV1/FVC (mean ± SD)	Serum ferritin (ng/dL) (mean ± SD)
Normal	10	85.1 ± 5.607	83.2 ± 5.432	101.7 ± 10.242	1329.7 ± 153.404
Mild	42	78.79 ± 5.068	74.24 ± 3.675	107 ± 5.81	3344.55 ± 704.119
Moderate	15	70.73 ± 5.133	65.13 ± 3.796	107.4 ± 9.709	5657.07 ± 426.934
Moderately severe	8	57.75 ± 7.555	55.88 ± 2.295	101.25 ± 12.748	5165.5 ± 1617.564
Severe	5	55 ± 9.695	49.2 ± 1.643	111.6 ± 18.515	8024.2 ± 588.643

The prevalence of the restrictive type of lung dysfunction according to this study is 875 per 1000 population. The prevalence calculated for varying severity of restrictive lung diseases was 525 per 1000 population, 187.5 per 1000, 100 per 1000 and 62.5 per 1000 population for mild, moderate, moderately severe and severe restrictive lung dysfunction, respectively, in thalassemia major patients.

The mean FEV1 observed was 85.1%, 78.7%, 70.73%, 57.75%, 55% respectively, in children with normal LFT, mild, moderate, moderately severe and very severe restriction of LFT as depicted in Table 2.

The mean FVC% observed was 83.2%, 74.24%, 65.13%, 55.88% and 49.2% respectively, in children with normal LFT, mild, moderate, moderately severe and very severe restriction of LFT (Table 2).

The mean FEV1/FVC% was >80% in all participants with lung involvement, thus ruling out obstructive lung disease. The pattern of PFT observed was of restrictive lung involvement with mean FEV1/FVC ratio of 107, 107.4, 101.25, 111.6 in children with normal PFT, mild, moderate, moderately severe and very severe restriction of LFT, respectively (Table 2).

The mean serum ferritin (ng/mL) levels corresponding to varying severities of restrictive lung disease were as follows: 1329.7 ng/mL in children with normal lung function, and 3344.55 ng/mL, 5657.07 ng/mL, 5165.5 ng/mL, and 8024.2 ng/mL in those with mild, moderate, moderately severe, and very severe restriction of lung function, respectively, as shown in Table 2.

**Figure 1:**
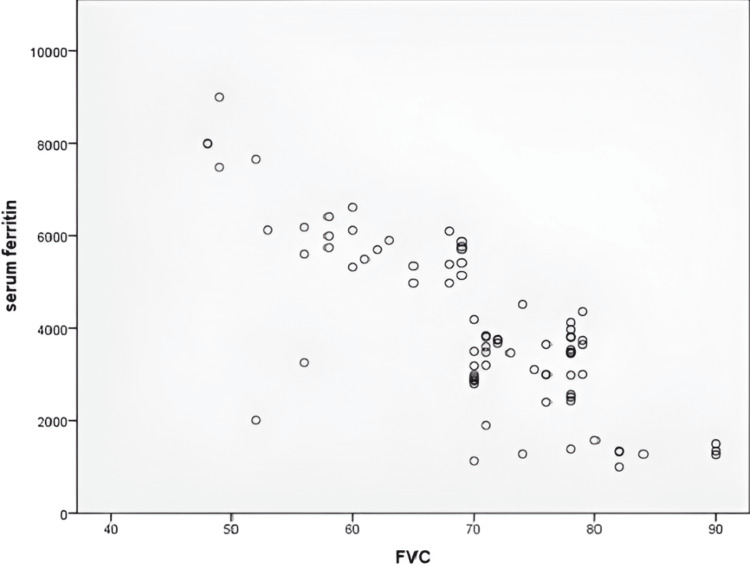
Scatter plot showing the negative correlation between FVC% and serum ferritin (ng/mL) (r = -0.789, p < 0.001) FVC%: forced vital capacity percentage

In this study, an inverse relationship was observed between serum ferritin levels and FVC%, with a strong negative correlation (r = -0.789) between the two variables, as determined by Pearson correlation, which was statistically significant, with a p-value of <0.001, as shown in Figure 1.

A positive correlation was observed between lung function and the total number of blood transfusions, with a correlation coefficient of r = 0.739 and a p-value of 0.000, indicating that the correlation is statistically significant (p < 0.001).

A negative correlation was found between FVC and height (r = -0.555) as well as between FVC and age (r = -0.630) in the sample, with a p-value of 0.000 for both, indicating statistically significant correlations (p < 0.001).

No significant correlation was found between lung function and pre-transfusion haemoglobin (r = 0.131, p = 0.246) and between serum ferritin and age at start of chelation (r = 0.157, p = 0.163). All children included in this study were on oral iron chelation.

## Discussion

Lung involvement is a known sequela of iron overload in thalassemia major patients. Over the past decades, research has shown various lung function abnormalities in children with thalassemia major. This study was aimed at assessing the lung dysfunction in children with thalassemia major and to find its correlation with iron overload using the status of serum iron. The secondary objectives were to find the prevalence of pulmonary dysfunction and the type and severity of lung dysfunction in children with thalassemia major.

Of the 80 children included in this study, 87.5% (n = 70) showed a restrictive pattern of lung involvement. All the children having lung involvement had an FEV1/FVC of 70% (0.70), thus ruling out an obstructive pattern of lung involvement. Our findings are similar to the study results of Gadiparthi et al. [14]. This pattern of lung dysfunction was similar to previous studies [2,5,6,8,15,16] where most patients had thalassemia major.

In the present study, a significant negative correlation was observed between serum ferritin and FVC%, which was statistically significant. The mean serum ferritin values (ng/mL) found in varying severity of restrictive lung diseases were 1329.7 ng/mL in normal subjects, 3344.55 ng/mL, 5657.07 ng/mL, 5165.5 ng/mL, 8024.2 ng/mL in children with mild, moderate, moderately severe and very severe restriction of PFT, respectively. This was similar to the study conducted by a few other studies done in India [6,15,17,18].

A similar statistically significant negative correlation between lung dysfunction and serum ferritin has been stated by other authors, such as Boddu et al. [2], Guidotti et al. [3], Rahim et al. [5] and Kandhari et al. [6], while no correlation between lung dysfunction and iron overload was found by Harsoor et al. [15].

In this study, we found a significant correlation between pulmonary impairment and the total number of blood transfusions, as well as the height and age of the children. These findings are consistent with those reported by Harsoor et al. [15], Ahmed et al. [16], Parakh et al. [8], Shivaswamy et al. [17], and Gadiparthi et al. [14]. However, Baruah and Bhattacharjee [18] did not observe any correlation with age or height.

This study adds that a restrictive pattern of lung dysfunction is associated with iron overload in children with thalassemia major, which correlates with the age, height and total number of blood transfusions received by the children. The limitations of our study include a small sample size, the unavailability of diffusion capacity and TLC measurements, and the single-centre study design. In order to overcome these limitations, a multi-centric study with a larger sample size evaluating all the parameters of lung function or a case-control study with comparison of lung function might be beneficial.

## Conclusions

In this study, we have found varying severity of restrictive lung dysfunction in children with thalassemia major despite regular iron chelation. There was a significant correlation between the severity of lung dysfunction and iron overload, and also a significant association between the total number of blood transfusions and serum ferritin. Hence, we conclude that children receiving multiple blood transfusions, even while on regular iron chelation, should undergo regular monitoring of PFTs, as it is a simple, cost-effective, and more importantly, non-invasive method. This makes it a better modality for assessing iron overload-related damage and guiding timely intensification of chelation therapy. PFTs should also be included as part of routine monitoring, as derangement in pulmonary function may occur with minimal or even no clinical symptoms. Therefore, timely monitoring and appropriate adjustment of iron chelation therapy may help improve the quality of life in patients with thalassemia.
